# Female Hygiene and Female Diseases

**Published:** 1884-08

**Authors:** 


**Female Hygiene and Female Diseases.** By J. K. Shirk, M. D. Lancaster Publishing Company. 1884.

This little book of about one hundred pages is intended for the instruction of women. So far as we have examined it, the book seems worthy of commendation as one which contains much good advice and instruction.

